# Barriers to tuberculosis treatment adherence in high-burden tuberculosis settings in Ashanti region, Ghana: a qualitative study from patient’s perspective

**DOI:** 10.1186/s12889-023-16259-6

**Published:** 2023-07-10

**Authors:** Maxwell Afranie Appiah, Joshua Appiah Arthur, Delphine Gborgblorvor, Emmanuel Asampong, Gideon Kye-Duodu, Edward Mberu Kamau, Phyllis Dako-Gyeke

**Affiliations:** 1 District Health Directorate, Ghana Health Service, Obuasi East, Ashanti Region Ghana; 2 Komfo Anokye Teaching Hospital, Kumasi, Ashanti Region Ghana; 3 School of Public Health, Greater Accra Region, University of Ghana, Legon, Ghana; 4 School of Public Health, University of Health and Allied Sciences, Ho, Volta Region Ghana; 5 UNICEF/UNDP/World Bank/WHO Special Programme for Research and Training in Tropical Diseases (TDR) at World Health Organisation, Geneva, Switzerland

**Keywords:** Tuberculosis, Patient-related barriers, Treatment adherence, Ashanti region, Ghana

## Abstract

**Background:**

Despite having an effective community-based Directly Observed Therapy Short-course (DOTS) strategy for tuberculosis (TB) care, treatment adherence has been a major challenge in many developing countries including Ghana. Poor adherence results in discontinuity of treatment and leads to adverse treatment outcomes which pose an increased risk of drug resistance. This study explored barriers to TB treatment adherence and recommended potential patient-centered strategies to improve treatment adherence in two high-burden TB settings in the Ashanti region of Ghana.

**Methods:**

The study was conducted among TB patients who defaulted on treatment in the Obuasi Municipal and Obuasi East districts in the Ashanti region. A qualitative phenomenology approach was used to explore the barriers to TB treatment adherence. Purposive sampling was adopted to select study participants with different sociodemographic backgrounds and experiences with TB care. Eligible participants were selected by reviewing the medical records of patients from health facility TB registers (2019–2021). Sixty-one (61) TB patients met the eligibility criteria and were contacted via phone call. Out of the 61 patients, 20 were successfully reached and consented to participate. In-depth interviews were conducted with participants using a semi-structured interview guide. All interviews were audio recorded and transcribed verbatim. The transcripts were imported into Atlas.ti version 8.4 software and analyzed using thematic content analysis.

**Results:**

Food insecurity, cost of transportation to the treatment center, lack of family support, income insecurity, long distance to the treatment center, insufficient knowledge about TB, side effect of drugs, improvement in health after the intensive phase of the treatment regimen, and difficulty in accessing public transportation were the main co-occurring barriers to treatment adherence among the TB patients.

**Conclusion:**

The main barriers to TB treatment adherence identified in this study reveal major implementation gaps in the TB programme including gaps related to social support, food security, income security, knowledge, and proximity to treatment centers. Hence, to improve treatment adherence there is a need for the government and the National Tuberculosis Programme (NTP) to collaborate with different sectors to provide comprehensive health education, social and financial support as well as food aid to TB patients.

**Supplementary Information:**

The online version contains supplementary material available at 10.1186/s12889-023-16259-6.

## Introduction

Tuberculosis has plagued humanity for many years and continues to be a major global health problem with millions of people affected yearly [1, 2]. Globally, 9.9 million people were reported to have developed TB in the year 2020. Africa is the second highest TB endemic region in the world and contributes to about 25% of global TB incidence cases [3]. In Ghana, the second national TB survey revealed a national prevalence rate of 290 per 100,000 population which is about three times higher than the estimated 92 per 100,000 by the World Health Organization (WHO) [4].

Tuberculosis is curable and its transmission can be prevented by early identification and treatment of infected persons [5]. Although, the resurgence of the Human Immunodeficiency Virus has made TB management very challenging, ensuring treatment completion is very important to control and prevent relapse and secondary drug resistance to TB [5]. The WHO recommends a Stop TB Strategy based on DOTS aiming at ensuring that TB patients take a standard short course of chemotherapy under guided supervision [6]. TB patients are to obtain assistance throughout their treatment duration and are encouraged to complete treatment to be cured and prevent resistance to any of the available anti-tuberculosis drugs [6]. To enhance treatment management through DOTS, a community-based intervention was initiated to allow community volunteers, lay health workers, or preferably a family member to offer treatment support to TB patients at the community level [5].

Despite the effectiveness of the community-based DOTS strategy, treatment adherence has been a major challenge in many countries [7]. Failure to complete TB treatment results in adverse outcomes such as lost-to-follow-up, treatment failure, resistance to TB drugs, and deaths [8]. In Ghana, although there have been substantial improvements in TB treatment, the success rate remains below the 90% set target in the country’s 2015–2020 strategic plan [3, 4]. The magnitude of the problem of adverse treatment outcomes differs across settings within the country. Usually, districts with a high prevalence of TB record unacceptably high lost-to-follow-up and death rates [4]. In the Ashanti region, for instance, districts like Obuasi Municipal and Obuasi East have consistently reported high rates of lost-to-follow-up and TB deaths over the past decades. Low adherence to TB medication increases the risk of prolonging infectiousness, relapse, and development or amplification of drug-resistant TB [7, 8].

Previous studies in diverse social settings have enumerated some risk factors for low TB treatment adherence including stigma, poor socioeconomic status, inadequate knowledge of TB, and treatment side effect, difficulty accessing treatment, and poor communication between healthcare providers and patients [9–12]. However, in Ghana, studies exploring patient perspectives regarding barriers to TB treatment adherence in the Ashanti region, the second region with the highest TB incident rate, are few [13, 14]. Notably, the WHO emphasizes patient-centered approaches to TB care as a vital element of TB control efforts. These approaches consider a patient as a primary figure in the healthcare continuum and acknowledge the patient’s personal and social circumstances in delivery care [2]. This study explored the barriers to TB treatment adherence from the perspective of TB patients who have ever defaulted on treatment in the Ashanti region of Ghana.

## Methods

### Study setting

**Fig. 1:**
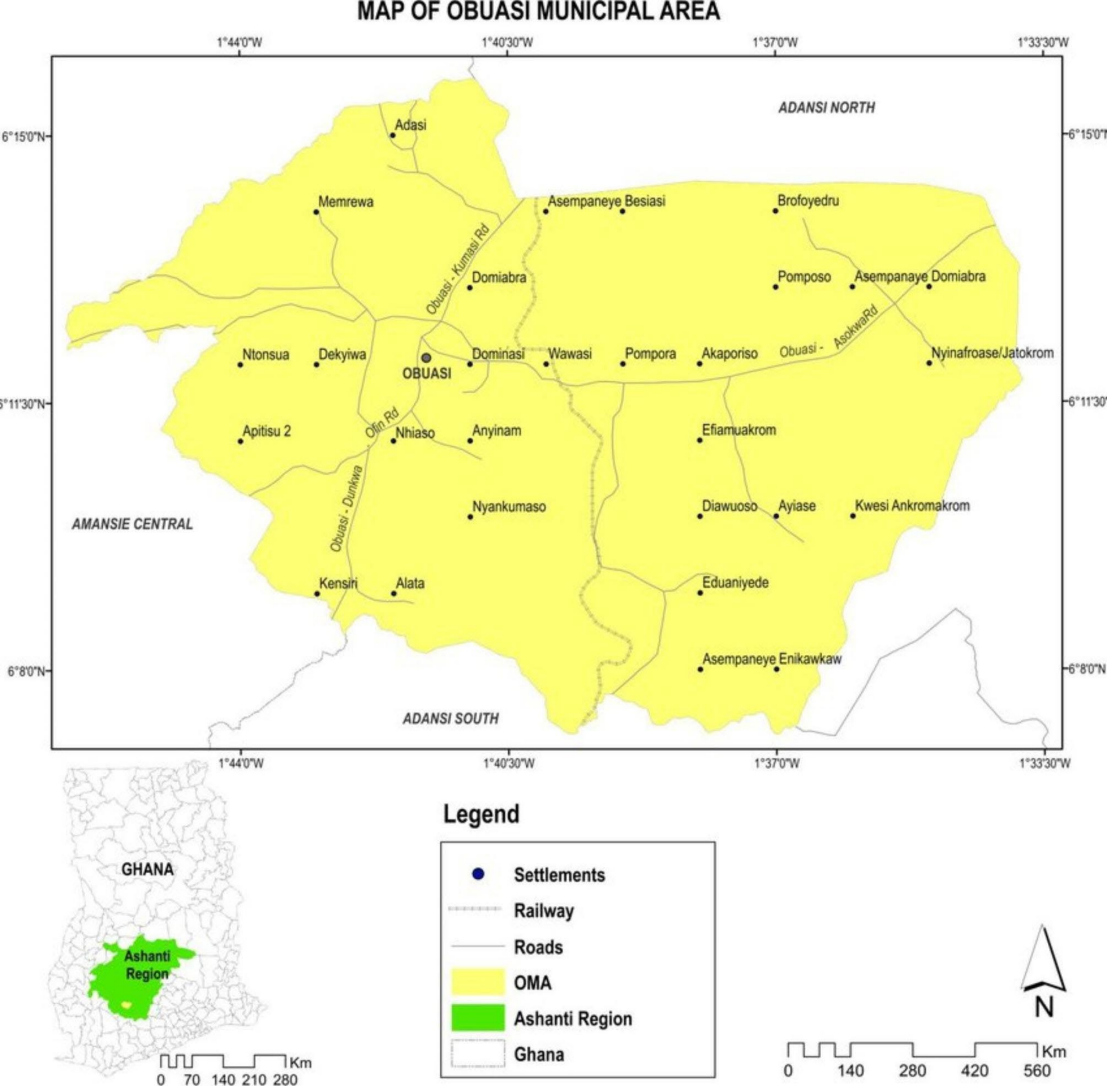
Combined geographical area of Obuasi Municipal and East district (formerly, Obuasi Municipal). The railway divides the two districts in Obuasi

This study was conducted in the Obuasi Municipal and Obuasi East district of the Ashanti region, Ghana. Formerly, both districts were one (Fig. 1) and were later split into two on 15th March 2018 [15]. Both districts are located in the southern part of the Ashanti region and cover a land area of 1624 km^2^, with a population size of 196,698 representing 3.6% of the region’s total population. They are bounded to the east by the Adansi South district, in the west by the Amansie Central district, and to the north by the Adansi North district. In 2021, according to the Ashanti Regional TB report, the case notification rate (CNR) of all forms of TB in both districts in Obuasi was 127.1 per 100,000 population (143.8 per 100,000 for Obuasi Municipal and 108 per 100,000 for Obuasi East district). This CNR was higher than the national average recorded in that year. Also, 72 (30.4%) of 237 TB patients enrolled in TB care in 2020 in both districts experienced adverse treatment outcomes with 55 (23.2%) lost to follow-up and 17 (7.2%) TB deaths [16].

### Study participants

The study included TB patients who had defaulted treatment from 2019 to 2021. Patients aged 18 years and above who have received a category I and III or Multidrug-resistant TB regimen for at least two weeks were considered eligible for inclusion. This age group was considered because it is the minimum age for providing informed consent under the 1992 Constitution of Ghana [17]. Patients who were seriously ill during the study period were excluded. Furthermore, TB patients receiving treatment in the two districts but living outside the districts were excluded from the study.

### Study design

A qualitative study, particularly, descriptive phenomenology was used to explore the barriers to TB treatment adherence. Descriptive phenomenology was chosen because it acknowledges that only individuals who have personally experienced a phenomenon can provide an accurate description of it as a lived experience [18, 19]. Thus, using phenomenology in this study allowed a better understanding and description of the personal views of patients’ experiences about their barriers to treatment adherence based on their different perspectives and background [20].

### Sampling

Purposive sampling method was used to select study participants with different sociodemographic backgrounds, remoteness, and experiences with TB care. The sample size was pre-defined based on a preliminary analysis of TB data which showed the number of TB defaulters in the study settings from 2019 to 2021. To achieve this, medical records of 746 TB patients were reviewed from the health facility TB registers and treatment folders in the three main hospitals which provide TB care in the study setting (that is, two hospitals from Obuasi East district and one from Obuasi Municipal). The review was done to identify the eligible participants (all patients aged 18 years and above who lived in the study setting) and their contact information. One-hundred and twenty-three (123) patients enrolled in TB care from 2019 to 2021 were identified to have defaulted treatment at the start of the study. However, sixty-one (61) of these defaulters met the inclusion criteria of the study and hence were pre-defined as study participants.

### Recruitment of participants

Patients’ information including telephone numbers was obtained from the health facility TB register of each hospital – a master register that contains demographics, results, and treatment outcomes of TB patients. Through the assistance of the TB DOT nurses, eligible patients were contacted via phone calls to inform them about the study. Patients who showed their willingness to participate were referred to the study team. The study team further informed interested patients about the purpose of the study and how the findings could potentially be used to improve TB care. After consenting to participate, each patient was scheduled for an interview. The patients were asked to choose where they feel appropriate to be interviewed. Patients’ choice of place for the interview was deemed very suitable since some may either choose the health facility or home or meet the interviewer at any place of convenience to feel at ease to provide information on their barriers to completing treatment.

### Data collection technique and tools

An in-depth interview (IDI) technique was used to gather data from the participants using a semi-structured interview guide. All participants were provided with an informed consent form to establish their consent before the interviews started. Information from the participants was audio-recorded. Four public health specialists were trained to assist in the data collection. The interview guide was adapted from existing literature [12] and modified to suit the study setting (Additional file 1). The interview guide was designed in English; however, interviews were conducted in the local dialect (Asante Twi) which is the most frequently used language among the people in the study setting. The interview guide was pretested randomly among five (5) TB patients at the TB treatment center of the Bryant Missions Hospital in Obuasi East district, to find out whether the questions were easily understood.

Each participant’s interview began with a general question, then the interviewer probed for more information based on the research questions. The general questions included, “Can you tell me about your experience as a TB patient?” and “What are the main challenges you faced during TB treatment?” (Additional file 1). All interviews were done face-to-face in different settings. The interviews lasted for a maximum of 30 min and were completed within a period of three months, that is, from, 19th July 2022 to 30th September 2022.

### Data analysis

All audio-recorded interviews were transcribed verbatim. The transcripts were repeatedly read to exclude any identifiable information and grammatical errors before analysis. The information gathered was cross-validated to ensure the information’s trustworthiness. During the cross-validation process, interview transcripts were read and translated into the local dialect for each participant to seek their approval and further clarification of any information. Additionally, investigator triangulation was used to reduce the risk of interviewer bias and improve the credibility and validity of the results. This was done by consistently discussing and reconciling the context of keywords, broader concepts, and units of meaning among the research team. The transcripts were imported into Atlas.ti version 8.4 software for data analysis. Similar responses were coded and grouped into major themes and sub-themes. Data was interpreted by analyzing the patterns of the codes among the participants. Potential relationships across the codes were examined through multiple occurring codes which overlap in a meaning unit. The final emerging codes, themes, and sub-themes were exported to Word for further interpretation of the data.

## Results

Sixty-one (61) TB defaulters were pre-defined for this study and all were contacted via phone call. Out of the 61 participants, 20 were successfully reached by the TB DOT nurses and consented to be interviewed, 8 were dead, 2 were seriously ill, and the telephone numbers of 31 participants could not be reached.

### Characteristics of participants

The age range of the TB patients was between 23 and 65 years. Most of these TB patients had Junior high school as their highest level of education and were unemployed. Additionally, the majority of the patients were not married, and only one participant was divorced (see additional details of participants’ characteristics in Additional file 2).

### Patient’s perspective on barriers to TB treatment adherence

The study identified six major themes, eight sub-themes, and thirteen codes from the patient’s perspective on barriers to TB treatment adherence. These themes were: (1) social and geographic; (2) economic; (3) knowledge and perception; (4) TB treatment; (5) nutrition; and (6) health service delivery. The summary of themes, sub-themes, and codes is shown in Table 1. The details of the code pattern and meaning units can be seen in Additional file 3. Several codes co-occurred in a meaning unit, suggesting a potential relationship across the codes. Nine main barriers were identified to have a potential relationship across codes, and these were food insecurity, cost of transportation, lack of family support, income insecurity, long distance to the treatment center, insufficient knowledge about TB, the side effect of drugs, improvement in health after the intensive phase of the treatment regimen, and difficulty in accessing public transportation. Data co-occurrence and potential relationships of the barriers can be seen in Additional file 4.

**Table 1:** Summary of Themes, Sub-themes, and Codes

Theme	Sub-theme	Codes
Social and geographic	Social	Family neglect, lack of family support, stigmatization
	Geographic	Long distance to the treatment center, difficulty in accessing public transport
Economic	Economic	Income insecurity, transportation cost
Knowledge and perception	Knowledge of TB	Insufficient knowledge of TB disease, drugs, and treatment duration
	Perception	Perception about the healthcare system
Treatment	TB treatment	Treatment side effects, improvement in health after the intensive phase of the treatment regimen
Nutrition	Feeding	Food insecurity
Healthcare service delivery	Healthcare service	Delay in laboratory services

### Social barriers – family neglect, lack of family support, and stigmatization

Family neglect was reported by some of the TB patients as a barrier to their completion of TB treatment. A patient indicated that none of his family members wanted to come near him because of the infectious nature of the disease, to the extent that his family members did not share the same drinking cup with him.

*“For this disease, if you get infected with it and you are in a family, nobody wants to come near you. They said it is contagious. Nobody even wants to drink from the same cup you drank from.” –* Male TB patient, 60 years old.

Another patient indicated that he expected his family members to help him by taking him to the hospital for his drugs, however, he never received a visit from any of them when he was diagnosed with TB.

*“None of my family members and loved ones paid me a visit for once*. *I expected them to help in taking me to the hospital so that I could complete my treatment. Because of that, I couldn’t do anything. I couldn’t even walk to the hospital so it made going for my medications from the hospital a very big problem for me” –* Male TB patient, 31 years old.

*‘When I fell sick, none of my family members have ever visited me.” –* Male TB patient, 38 years old.

Lack of family support was reported by some of the TB patients who defaulted treatment as a barrier to their treatment adherence. For instance, a patient indicated that his wife requested a divorce and left him because of his poor condition and inability to work to cater to his family, and thus he did not receive support henceforth.

*“Because I am unwell and so I am unable to work, my wife had to ask for a divorce since I could not get money to cater for my family, so I did not get anyone to support me from then.” –* Male TB patient, 52 years old.

Similarly, a patient mentioned that *“Currently, I am not working and my parents are no more so I have nobody to depend on. I have nobody to help me take my drugs.” –* Male TB patient, 41 years old.

Stigmatization was also identified by a TB patient who indicated that he behaved in a way such that he would not be noticed by people in the community that he had TB. He added that his close friend who knew his condition feared close contact with him and behaved in an unfriendly way toward him.

*“Because I heard that the disease was deadly, I did not behave in a way to make people notice that I had tuberculosis. It was only my family and close ones who knew. Despite the frequency of the cough, people did not know that I had tuberculosis. There is even a friend I usually would visit who knew I had tuberculosis. Due to the frequent coughing, she did not like me to get closer to her anytime I visited. She would tell me to go away with my cough, as well as say other things.” –* Male TB patient, 52 years old.

### Geographical barriers – long distance to the treatment center and difficulty in accessing public transport

Accessibility to a TB treatment center was a major hindrance to patients’ continuous adherence to TB treatment. Most of the patients reported long distances to the TB treatment center as a challenge to their continuous receiving of their monthly doses of TB drugs as per the TB guideline.

*“The distance to the hospital is far and the path is hilly so I am unable to climb the hills.” –* Male TB patient, 31 years old.

*“I stay far from the tuberculosis treatment center in Obuasi and had it not been for my sister who assisted me with my transportation, I would not have been able to come for my medications even up to the third month after which I stopped coming for the medication” –* Male TB patient 52 years old.

In addition to distance, some patients indicated transportation difficulty as a major geographical barrier. For example, a patient reported that accessing a vehicle from where he stays to the treatment center was sometimes a challenge.

*“I live at Ayaase [8.7km from health facility] and getting a car is sometimes difficult because of where I stay.” –* Male TB patient, 60 years old.

Another patient mentioned that due to transportation challenges community members walk from their homes to another community before having easy access to a vehicle, however, due to his poor condition, he is unable to walk.

*“Usually, it is hard to find a vehicle from coming into the community so most of us walk from Mampamhwe to Kwabenakwa Junction which is very far. Looking at my situation with this disease it is difficult to walk that far” –* Male TB patient, 41 years old.

Likewise, another patient mentioned that he walks from his community to the TB treatment center due to difficulty in accessing public transport and usually experiences tachypnea, as a result, he stopped visiting the hospital for his medication.

*“When I am coming to the hospital, I walk from Odumasi to Aboagyekrom and pass behind a school to Boete where Bryant Mission Hospital (a treatment center) is. It is very far. Walking to the Bryant Mission Hospital is what disturbs me, it makes my breathing rapid. Because I walk, I stopped taking the drugs after two months when the cough seized.” –* Male TB patient, 37 years old.

### Economic barriers – income insecurity and transportation cost

Generally, almost all the TB patients who defaulted on treatment reported income insecurity or financial problem as the main barrier to TB treatment adherence. To prevent the spread of TB, patients are required to stay at home to take their TB medicine until the healthcare provider says they can return to work. Consequently, some patients reported that they eventually lost their jobs, and experienced financial difficulty. Others also mentioned that they stopped their monthly visits to the hospital for their TB medicine since they were unable to work and get income.

*“I lost my job, and I have no one to take care of me and I find it difficult to get food to eat. Because I have no financial support, I left Obuasi to Wa because my family members were there and even if I cannot get money, I knew they will support me to eat*. *I did not go to any hospital for some time in Wa until when my condition worsened” –* Male TB patient, 32 years old.

*“I was put on medication and was asked to visit every month for my medication. However, because I was unwell and was not working, I had to stop paying my monthly visits to the hospital even though the medications were making me better. I had to stop because my money got finished and I did not get help from anyone”* – Male TB patient, 52 years old.

Additionally, a patient mentioned that he stopped taking the TB drugs after 3 months of treatment to return to Galamsey (illegal mining) due to household income insecurity.

*“I did not go to the hospital for my drugs because of hardship. After taking the drugs for 3 months, I stopped to do the ‘galamsey’ I used to do, to get money.” –* Male TB patient, 33 years old.

Another patient also reported that he became dependent on his brother, and was able to receive money for transportation to the hospital for his monthly TB medication as and when he got money from him.

*“It was my brother who was giving me money for transportation but lately because of financial constraints, he is not able to give me money to come for my medications when they get finished, so it is now that he has gone to borrow money for me to be able to come for my medications*.” – Male TB patient, 60 years old.

Cost of transportation was identified as another major barrier to patient adherence to TB treatment. The patients expressed difficulties in acquiring money for transportation fares, which caused delayed or interrupted treatment. Some patients reported that the transport fare from their abode to the treatment facility was high and this come along with the necessity for food during a visit. This situation led to their inconsistent adherence to treatment schedules.

*“Sometimes getting money for transport fare was difficult for me.” –* Male TB patient, 46 years old.

*“Money for feeding and transportation to the hospital are the reasons why I couldn’t complete my TB treatment*, *but money to pick a vehicle to the hospital was my main challenge.” –* Male TB patient, 31 years old.

*“If I take a car from Mampamhwe to the treatment center and back to the house, it is more than GH¢10. I hardly get such an amount of money and I surely cannot go to the hospital on an empty stomach. This is why I cannot visit the treatment center for my drugs according to schedule.” –* Male TB patient, 41 years old.

A patient expressed that if the drugs were offered closer to their home, it would reduce the burden of transportation costs.

*“Money for transportation is another problem. If the drugs are brought closer to me, it would help me and I will not complain of not having money for transport.” –* Male TB patient, 65 years old.

### Knowledge barriers – insufficient knowledge of TB disease, drugs, and treatment duration

Insufficient knowledge about TB, drugs, and treatment duration was identified as a barrier to the completion of treatment for some patients. A patient reported that he had no idea of the TB disease and its mode of transmission.

*“When I went to the hospital, I was only told the cough I am presenting with is a TB cough, but I have no idea of what the disease is. I don’t know how it is spread and how I even got it” –* Male TB patient, 41 years old.

Another patient mentioned that aside from being told how to take the TB drugs daily, he did not know about the TB drugs he received.

*“I have no idea of the drugs I am taking. What I have only been told is that when I wake up in the morning, I should brush my teeth and take the drug before eating” –* Male TB patient, 65 years old.

Additionally, a TB patient mentioned that he stopped treatment for a month because he did not know that after undergoing a medical review by a doctor during the course of his treatment, he was supposed to continue for 6 months.

*“…I did not know I was supposed to continue to take the drugs till the sixth month despite having seen the doctor, so for almost one month I never took any drug.” –* Male TB patient, 35 years old.

### Perception barrier – perception about the health care system

Fear of returning to the hospital after defaulting treatment due to patients’ negative perceived reaction from the healthcare provider was identified as a barrier to TB treatment adherence. A patient reported that he decided not to visit the hospital for his drugs when he defaulted because he feared that the healthcare providers would complain.

*“When I defaulted, I felt shy and feared to return to the hospital because I thought the health workers will be angry and complain that I did come for my drugs on time so I decided not to come at all.” –* Male TB patient, 37 years old.

### TB treatment – Treatment side effects and improvement in health after the intensive phase of the treatment regimen

TB Treatment side effect was reported by some TB patients as a barrier to treatment adherence. Tiredness and hunger resulting from the TB drugs were the most reported side effects that led to the patient’s refusal to adhere to treatment.

*“The medicine is very strong and makes you tired when you take it. So, I decided to stop taking it.” –* Male TB patient, 36 years old.

*“Taking the drugs makes me feel very hungry so I decided to stop taking them because I would not get money for food.” –* Male TB patient, 52 years old.

This study also identified improvement in health after the intensive phase of the treatment regimen as a barrier to TB treatment adherence. That is, some TB patients declared themselves fit after taking the drugs for some time and thus decided to stop taking the TB drugs without any medical advice.

*“No one permitted me to stop taking the drugs. I stopped taking them myself because I realized I was fit after taking the drugs for 3 months. My brother does not even know I have stopped taking the medications.” –* Male TB patient, 36 years old.

### Nutrition – food insecurity

This study identified issues related to food insecurity as a major barrier to TB treatment adherence among TB patients. Some of the patients reported hunger due to financial constraints as their reason for not adhering to the TB medication.

*“It is the hunger I was going through that made me stop taking the drugs. What I will use to pick a car to the hospital for my drugs, I will rather use it to buy food.” –* Male TB patient, 33 years old.

*“I have nothing to buy food to eat so even in my illness I support a neighbour to unpack luggage so I can get money to buy food” –* Male TB patient, 38 years old.

*“When I take the drugs, I become very hungry. I was able to take half of the drug in the first month that I started but I couldn’t continue because I did not get food to eat and support.” –* Male TB patient, 32 years old.

Other patients also mentioned that based on the counsel they received from the healthcare providers, they are supposed to take the drugs minutes/hours before eating, however, the TB medicine makes them feel very hungry whenever they take it, so they only take the drug when they are sure they will get food to eat.

*“They usually gave me drugs within two weeks intervals so when I start and it is left with about four or five days for it to finish and I don’t have money to feed, then I break, so when in two days I get money then I drink it, and when it is finished, I go for continuation from the hospital. “–* Male TB patient, 37 years old.

*“I have to take the drugs 30 minutes before I eat and so for instance, if I take the drugs and then take porridge in the morning, after a while if I do not get food to eat, I feel very hungry*. *So, I could not complete my treatment due to hunger from taking the drugs.” –* Male TB patient, 52 years old.

*“They said when I take the drug, I should wait for one hour before I eat. So, at a point when I don’t have money to buy food, I don’t take the drug. I only take the drug only when I know that I have gotten money to buy food to eat” –* Male TB patient, 65 years old.

### Healthcare service delivery barrier – delay in laboratory services

A delay in laboratory service was also identified by a patient as a barrier to his adherence to TB treatment. The patient reported that he paid several visits to the hospital for his laboratory report so that he can be counseled on the progress of his health while on treatment, but he never got the laboratory report, therefore he decided to stop taking the TB drugs.

*“I was told to go to the lab to do a test and when the report comes, they will see whether or not I will be allowed to continue treatment. The lab man told me to come in a week for my report but when I went, I was told the report was not ready. I went home and returned to the hospital for my report and this I did for one month, but I still did not get the report. So, I decided to stop taking the drugs. I didn’t go to see the TB nurse again for any drugs” –* Male TB patient, 65 years old.

## Discussion

This study explored the barriers to TB treatment adherence from the patient’s perspective in the Ghanaian setting and found several related barriers. These were food insecurity, cost of transportation to the treatment center, lack of family support, income insecurity, long distance to the treatment center, insufficient knowledge about TB, the side effect of drugs, improvement in health after the intensive phase of the treatment regimen, and difficulty in accessing public transportation.

Support systems have been noted as a protective factor for treatment adherence while a lack of such support is a major hindrance [21]. The finding of this study showed a lack of family support to be a major barrier to TB treatment adherence. The majority of patients in this study expressed grave concerns regarding the lack of support of any sort, especially from family members, to continue their treatment. This finding agrees with studies in Eritrea [21] and Indonesia [12]. Some patients emphasized that if they had adequate support they could have continued or completed their treatment. This suggests that patients who have little social support, like some elderly or the poor, may not benefit from effective health interventions such as free access to TB medications [21].

This study also found stigmatization as another barrier to TB treatment adherence. This finding is consistent with studies in Indonesia [12, 22], which indicated that people with TB still carry a social stigma. Interestingly, the stigma in this study originated from family members and close friends, which subsequently led to neglect among some patients. Emphasizing family neglect, some patients reported that none of their family members wanted to come near or visit them, to the extent of refusing to share common household items with them. This study further revealed that some patients were discouraged to visit a treatment center for their medication as scheduled because of the long distance. Some patients had to go through so much stress walking for long hours to reach a treatment center for care, and as such refused or delayed treatment. This finding agrees with studies in Ethiopia [23] and Indonesia [12]. Geographic access to healthcare services is an important issue and well-studied [12, 21, 23]. As shown in this study, long distance to the nearest health center for DOTS presents a significant challenge to many patients, coupled with the absence of comprehensive and affordable transport services.

This study identified income insecurity as a major economic barrier to TB treatment adherence. A similar finding was reported in studies in Eritrea [21], Indonesia [12], and Ethiopia [23]. Being sick means that patients can lose their jobs or be unable to keep working if self-employed. This is coupled with attendant financial costs such as the cost of transport and food even though TB drugs are free [24, 25]. These concerns were expressed by the majority of the patients in this study who indicated that their inability to access food and pay for transport costs was mainly due to income insecurity. Additionally, TB drugs demand patients to consume extra food, especially protein-rich foods to restore their health, which often goes beyond their financial ability [26]. This situation was notably expressed by most TB patients in this study who noted that the TB medicine made them feel very hungry, so they only took the drug when they were sure of getting food to eat. This finding agrees with studies in South Africa [27], and Eritrea [21].

Insufficient knowledge about TB and negative perception of the health care system were found as barriers to TB treatment adherence among the TB treatment defaulters. Some of the patients did not know about the disease they were suffering from, its mode of transmission, and the TB drugs they were taking. A number of them did not know that the standard treatment duration for TB was 6 months and the consequences they would face if they stopped taking the drugs. This finding agrees with previous studies in Ghana [13], Indonesia [12], Eritrea [21], and Pakistan [28]. The finding of this study also showed that a patient felt shy and feared returning to the TB treatment center after defaulting treatment with a perception that healthcare workers in charge of TB management would be fed up and complain. This negative perception could be a major reason why TB patients visit different health facilities for care aside from stigma. This finding also suggests that TB patients were not provided with enough health education and counseling before taking their medication. Studies have found that educating a patient significantly reduces the risk of treatment non-adherence [29, 30]. Thus, healthcare providers should be trained and encouraged to provide more personalized health education on TB within the context of the patient’s background and local customs [21].

This study found treatment side effects as another barrier to TB treatment adherence. Responses from some patients indicated that experiences of TB drug side effects led to their refusal to adhere to the treatment. This could be because some patients believed that the TB drugs worsen their condition, despite having the capacity to cure them [21]. Similar studies have been reported in Ghana [13], Eritrea [21], and Indonesia [12]. Excessive hunger and weakness were the most experienced side effect which deterred some patients to discontinue their treatment in this study. It is suggested that patients should be informed of the common side effect they should anticipate and what to do once they occur.

### Limitations of the study

There was a challenge in reaching some patients who had defaulted treatment which led to a reduction in the pre-defined sample size. Telephone numbers of some of the TB defaulters could not be reached, hence we could not ascertain whether or not they were still alive. Despite this challenge, the desired sample size accepted for qualitative research was reached. Additionally, all participants who were reached and agreed to participate were males. Thus, the sample is not gender representative and so the findings are likely to miss out on key gendered factors/experiences in non-adherence to TB management. Again, though the findings are instructive, they are not meant to be generalizable because it is a qualitative study. The findings can form the basis of a more representative population study to study the presence or otherwise of the key factors identified through this study. Thus, further quantitative study is needed to generalize the results to the population. Furthermore, as situations differ per geographical location a nationwide study should be conducted to broaden the current understanding of the barriers to TB treatment adherence as well as identify potential relationships between the barriers.

### Study implication

The barriers to TB treatment adherence identified in this study reveal major implementation gaps in the TB programme which can lead to a surge in drug-resistance TB if not addressed. These gaps include a lack of social support, food insecurity, income insecurity, poor knowledge, and increased travel distance to treatment centers.

This study revealed a potential relationship between income insecurity, food insecurity, and lack of family support and TB treatment outcomes. Addressing the welfare challenges of some TB patients who default is key to ensuring that they adhere to their medications. Therefore, efforts at addressing poor adherence should include an identification of economically vulnerable patients and the institution of some financial support throughout the treatment regime. More sustainably, such efforts could be routed through existing poverty alleviation programs already in place at the local government level such as LEAP [21, 31]. This could address the issue of lost-to-follow-up and reduce the mortality rate among TB patients resulting from patients’ financial limitations.

Educating patients and the community on TB disease, prevention, care, and management is vital to treatment adherence. TB patients should be counseled on the importance of TB drugs, the anticipated side effects, and the consequences they may incur for themselves and the community if they refuse to adhere to treatment. The public should be educated on the need to avoid stigma. Additionally, healthcare providers should provide psychosocial support to TB patients through counseling to mitigate any potential psychological problem from stigma.

Furthermore, this study revealed that long distance to TB treatment centers is related to transportation issues, especially for TB patients in far-to-reach communities. Some participants’ thoughts during the interview suggest that when TB drugs are brought closer to them this problem may be solved. Although policy in Ghana advocates for the inclusion of CHPS compounds and community pharmacies in TB treatment, this implementation is yet to be fully optimized in many districts in the country. Thus, we highly recommend that the Ministry of Health and NTP optimize the implementation of this policy at all levels, to address the transportation-related barriers that hinder the delivery of TB treatment. Moreover, home visits where drugs are delivered to clients by healthcare service providers should be encouraged, particularly for patients who are at risk of treatment default. These approaches bring the treatment directly to the patients, making it more convenient and reducing the likelihood of treatment interruption and discontinuation [9, 32].

Considering the influence of TB drug side effects on treatment adherence, a strategy to address this issue could be to involve pharmacists for direct patient service in TB management to act as treatment supporters who educate, monitor, and evaluate drug use based on the principles of pharmaceutical care [12]. Previous studies have reported that pharmacists’ direct involvement in TB patients’ management improved treatment success [33, 34].

## Conclusion

This study showed food insecurity, cost of transportation to a treatment center, lack of family support, income insecurity, long distance to the treatment center, insufficient knowledge about TB, the side effect of drugs, improvement in health after the intensive phase of the treatment regimen, and difficulty in accessing public transportation as the main barriers to TB treatment adherence. To enhance treatment adherence, there is a need for the government and NTP to collaborate with different sectors and initiatives to provide comprehensive health education, social and financial support, as well as food aid to TB patients.

## Electronic supplementary material

Below is the link to the electronic supplementary material.

**Additional file 1**. Patient-focused interview

**Additional file 2**. Characteristics of study participants

**Additional file 3**. Code patterns

**Additional file 4**. Co-occurrence of data and potential relationships

## Data Availability

The datasets used and/or analyzed during the current study are available from the corresponding author on reasonable request.

## List of abbreviations

CHPS: Community-based Health Planning and Services
DOTS: Direct Observed Treatment Short course
GHS-ERC: Ghana Health Service Ethical Review Committee
IDI: In-depth Interview
NTP: National Tuberculosis Control Programme
TB: Tuberculosis
WHO: World Health Organization
