# Supplementary material for: Barriers to tuberculosis treatment adherence in high-burden tuberculosis settings in Ashanti region, Ghana: a qualitative study from patient’s perspective
Source: BMC Public Health. 2023 Jul 10;23:1317. doi: 10.1186/s12889-023-16259-6 (PMC10332032; doi:10.1186/s12889-023-16259-6)
Supplement: Supplementary file 1 — Additional file 1. Patient-focused interview [file 12889_2023_16259_MOESM1_ESM.pdf]

Additional File 1: Patient-Focused Interview

DATA COLLECTION TOOL

Socio-demographic Characteristics

1. Age (in years) ________________
2. Gender? A. Male [ ] B. Female [ ]
3. Marital status? A. Single [ ] B. Married [ ] C. Divorced [ ] D. Widowed [ ]
4. Educational status? A. No formal education [ ] B. Basic education [ ] C. JHS [ ]
   D. SHS [ ] E. Tertiary [ ]
5. Occupation ____________________
6. Place of interview. A. Home [ ] B. Hospital [ ] C. Others (Specify) ________________

INTERVIEW GUIDE FOR TB PATIENT

GENERAL QUESTION (ASK ANY OF THESE QUESTION FIRST)

Can you tell me about your experience as a TB patient?

Why are you not able to complete your TB treatment?

What are the main problems you faced during TB treatment?

PROBING QUESTIONS (ASK ONLY WHEN YOU NEED TO PROBE)

Socio-demography and economic status

How do your family and others support you?

What do families/others need to do to help in the treatment?

How does distance affect the treatment?

How does the availability and cost of transportation affect your treatment?

How does your income affect your treatment?

What would say about the cost of treatment at private /public?

Knowledge and perception

Tell me all you know about TB?

Before your treatment what did the TB-DOT nurse tell you?

What do people say or perceive about someone who has TB?

TB treatment

How does taking of the drugs go?

What can you say about the drug and it taking?

What can you say about the duration of the treatment?

What challenges do you have face that deter you from taking the drugs?

Can you tell me what could be done to improve upon the treatment of TB?
