# Supplementary material for: Barriers to tuberculosis treatment adherence in high-burden tuberculosis settings in Ashanti region, Ghana: a qualitative study from patient’s perspective
Source: BMC Public Health. 2023 Jul 10;23:1317. doi: 10.1186/s12889-023-16259-6 (PMC10332032; doi:10.1186/s12889-023-16259-6)
Supplement: Supplementary file 2 — Additional file 2. Characteristics of study participants [file 12889_2023_16259_MOESM2_ESM.pdf]

Additional file 2

Characteristics of study participants

Participants characteristics	Frequency (n=20)	Percentage (%)
**Age (years)**		
<30	2	10.0
30 to 40	12	60.0
>40	6	30.0
**Gender**		
Male	20	100.0
Female	0	0
**Educational level**		
Junior high school	9	45.0
Senior high school	8	40.0
Tertiary	3	15.0
**Marital status**		
Married	5	25.0
Single	10	50.0
Divorced	1	5.0
Widowed	4	20.0
**Means of interview**		
Home	16	80.0
Hospital	1	5.0
Street	1	5.0
Telephone	2	10.0
**Occupation**		
Driver	1	5.0
Farmer	4	20.0
Mechanics	1	5.0
Student	1	5.0
Trader	1	5.0
Unemployed	12	60.0
