# Supplementary material for: Barriers to tuberculosis treatment adherence in high-burden tuberculosis settings in Ashanti region, Ghana: a qualitative study from patient’s perspective
Source: BMC Public Health. 2023 Jul 10;23:1317. doi: 10.1186/s12889-023-16259-6 (PMC10332032; doi:10.1186/s12889-023-16259-6)
Supplement: Supplementary file 3 — Additional file 3. Code patterns [file 12889_2023_16259_MOESM3_ESM.pdf]

Additional file 3: Code Patterns

Coding process of implementation problems identified

**Meaning Unit**	**Codes**	**Sub-themes**	**Themes**
"Because I heard that the disease was a deadly disease, I did not behave in a way to make people notice that I had tuberculosis. It was only my family and close ones who knew. Despite the frequency of the cough, people did not know that I had tuberculosis. There is even a friend I usually would visit who knew I had tuberculosis. Due to the frequent coughing, she did not like me to get closer to her anytime I visited. She would tell me to go away with my cough, as well as say other things." – Male TB patient, 52 years old.	Stigmatization	Social barrier	Social and geographic
"For this disease, if you get infected with it and you are in a family, nobody wants to come near you. They said it is contagious. Nobody even wants to drink from the same cup you drank from." – Male TB patient, 60 years old	Family neglect	Social barrier	
"None of my family members and loved ones paid me a visit for once. I expected them to help in taking me to the hospital so that I could complete my treatment. Because of that, I couldn't do anything. I couldn't even walk to the hospital so it made going for my medications from the hospital a very big problem for me" – Male TB patient, 31 years old	Family neglect	Social barrier	
'When I fell sick, none of my family members have ever visited me." – Male TB patient, 38 years	Family neglect	Social barrier	
"Because I am unwell and so I am unable to work, my wife had to ask for a divorce since I could not get money to cater for my family, so I did not get anyone to support henceforth." – Male TB patient, 52 years old	Family support	Social barrier	
"As I am here should I say I am not going through hardship then it is a lie. Currently I am not working and my parents are no more so I have nobody to depend on. I have nobody to help me take my drugs." – Male TB patient, 41 years old	Family support	Social barrier	
"I have a grandson with me but he is always not around to support me to take my drugs. Sometimes I don't find anybody to run errand for me so I become hungry and delay	Family support	Social barrier	

or not take the drugs. The support I have is not enough" – Male TB patient, 65 years old			
"I do not have anybody to really help me and that is one of the main reasons why I have not been able to come for my medications." – Male TB patient, 52 years old	Family support	Social barrier	
"I live at Ayaase and getting a car is sometimes difficult because of where I stay." – Male TB patient, 60 years old.	Transportation difficulty	Geographical barrier	
"Usually, it is hard to find a vehicle from coming into the community so most of us walk from Mampamhwe to Kwabenakwa Junction which is very far. Looking at my situation with this disease it is difficult to walk that far" – Male TB patient, 41 years old	Transportation difficulty	Geographical barrier	
"I stay far from the tuberculosis treatment centre in Obuasi and had it not been for my sister who assisted me with my transportation, I would not have been able to come for my medications even up to the third month after which I stopped coming for the medication" – Male TB patient 52 years old	Long distance	Geographical barrier	
"The distance to the hospital is far and the path is hilly so I am unable to climb the hills." – Male TB patient, 31 years old	Long distance	Geographical barrier	
"When I am coming to the hospital, I walk from Odumasi to Aboagyekrom, and pass behind a school to Boete where Bryant Mission Hospital is. It is very far. Walking to the treatment center is what disturbs me, it makes my breathe becomes rapid. Because I walk, I stopped taking the drugs after two months when the cough seized." – Male TB patient, 37 years old.	Long distance	Geographical barrier	
"Because I have no financial support, I left Obuasi to Wa because my family members were there and even if I cannot get money, I knew they will support me to eat. I did not go to any hospital for some time in Wa until when my condition worsened" – Male TB patient, 32 years old.	Income insecurity	Economic barrier	Economic
"I was put on medication and was asked to visit every month for my medication. However, because I was unwell and was not working, I had to stop paying my monthly visits to the hospital despite the fact that the medications were making me better." – Male TB patient, 52 years old	Income insecurity	Economic barrier	

"It was my brother who was giving me money for transportation but of late, because of financial constraints, he is not able to give me money to come for my medications when they get finished, so it is now that he has gone to borrow money for me to be able to come for my medications." – Male TB patient, 60 years old	Income insecurity	Economic barrier	
"I did not go to the hospital for my drugs because of hardship. After taking the drugs for 3 months, I stopped to do the 'galamsey' to I used to do to get money." – Male TB patient, 33 years old	Income insecurity	Economic barrier	
"Sometimes getting money for transport fare was difficult for me." – Male TB patient, 46 years old.	Transportation cost	Economic barrier	
"I live alone at Obuasi and do not have anyone to help me so I had to leave to my hometown and only went back to the hospital whenever my medications got finished for another set of medication. However, it got to a time where all my money got finished, so I did not get money to take transport to the hospital." – Male TB patient, 60 years old	Transportation cost	Economic barrier	
"Money for food insecurity and transportation to the hospital are the reasons why I couldn't complete my TB treatment, but money pick a vehicle to the hospital was my main challenge." – Male TB patient, 31 years old	Transportation cost	Economic barrier	
"Money for transportation is another problem. If the drugs are brought closer to me, it would help me and I will not complain of not having money for transport." – Male TB patient, 65 years old	Transportation cost	Economic barrier	
"You will feel the transportation fare to the hospital is little but even what to use to buy porridge is difficult. Money to take transport is a problem." – Male TB patient, 33 years old	Transportation cost	Economic barrier	
"From Kwabenakwa Junction to Mampamhwe where I stay is GH2.50. Taking vehicle back and forth will be GH5.00. Now from Kwabenakwa Junction to Boete is GH1.80 and from Boete to the hospital is GH1.50. If I take a care from Mampamhwe to the treatment center and back to the house	Transportation cost	Economic barrier	

is more than GH10. I hardly get such an amount of money and I surely cannot go to the hospital on empty stomach. This is why I cannot visit the treatment center for my drugs according to schedule." – Male TB patient, 41 years			
"…I did not know I was supposed to continue to take the drugs till the sixth month despite having seen the doctor, so for almost one month I never took any drug." – Male TB patient, 35 years old	Knowledge of TB treatment	Knowledge barrier	Knowledge and perception
"I have no idea of the drugs I am taking. What I have only been told is that when I wake up in the morning, I should brush my teeth and take the drug before eating" – Male TB patient, 65 years old	Knowledge of TB treatment	Knowledge barrier	
"When I went to the hospital, I was only told the cough I am presenting is TB cough, but I have no idea of what the disease is. I don't know how it is spread and how I even got it" – Male TB patient, 41 years old	Knowledge of TB disease	Knowledge barrier	
"When I defaulted, I felt shy and feared to return to the hospital because I thought the health workers will be bored and complain that I did come for my drugs on time so I decided not to come at all." – Male TB patient, 37 years old	Perception about health care system	Perception barrier	
"The medicine is very strong and makes you tired when you take it. So, I decided to stop taking it." – Male TB patient, 36 years old	Side effect of drug	Side effect of drug	TB treatment
"Taking the drugs makes me feel very hungry so I decided to stop taking them because I would not get money for food." – Male TB patient, 52 years old	Side effect of drug	Side effect of drug	
"No one permitted me to stop taking the drugs. I stopped taking them myself because I realized I was fit after taking the drugs for 3 months. My brother does not even know I have stopped taking the medications." – Male TB patient, 36 years old.	Improvement in health after the intensive phase of the treatment regimen	Improvement in health after the intensive phase of the treatment regimen	
"I have to take the drugs 30 minutes before I eat and so for instance, if I take the drugs and then take porridge in the morning, after a while if I do not get food to eat, I feel very hungry. So, I could not complete my treatment due to hunger from taking the drugs." – Male TB patient, 52 years old	Food insecurity	Food insecurity	Nutrition

"They said when I take the drug, I should wait for one hour before I eat. So, at a point when I don't have money to buy food, I don't take the drug. I only take the drug only when I know that I have gotten money to buy food to eat" – Male TB patient, 65 years old	Food insecurity	Food insecurity	
"They usually gave me drugs within two weeks interval so when I start and its left with about four or five days for it finish and I don't have money to feed, then I break, so when in two days' time I get money then I drink it, and when its finished I go for continuation from the hospital. "– Male TB patient, 37 years old	Food insecurity	Food insecurity	
"It is the hunger I was going through that made me stop. What I will use to pick car to the hospital for my drugs, I will rather use it to buy food." – Male TB patient, 33 years old	Food insecurity	Food insecurity	
"I have nothing to buy food to eat so even in my illness I support a neighbour to unpack luggage so I can get money to buy food" – Male TB patient, 38 years old	Food insecurity	Food insecurity	
"I lost my job, and I have no one to take care of me and I find it difficult to get food to eat. When I take the drugs, I become very hungry. I was able to take half of the drug in the first month that I started but I couldn't continue because I did not get food to eat and support." – Male TB patient, 32 years old	Food insecurity	Food insecurity	
"I was told to go to the lab to do a test and when the report comes, they will see whether or not I will be allowed to continue treatment. The lab man told me to come in a week time for my report but when I went, I was told the report was not ready. I went home and returned to the hospital for my report and this I did for one month, but I still did not get the report. So, I decided to stop taking the drugs. I didn't go to see the TB nurse again for any drugs" – Male TB patient, 65 years old	Delay in laboratory services	Delay in laboratory services	Healthcare service delivery
