# Supplementary material for: Barriers to tuberculosis treatment adherence in high-burden tuberculosis settings in Ashanti region, Ghana: a qualitative study from patient’s perspective
Source: BMC Public Health. 2023 Jul 10;23:1317. doi: 10.1186/s12889-023-16259-6 (PMC10332032; doi:10.1186/s12889-023-16259-6)
Supplement: Supplementary file 4 — Additional file 4. Co-occurrence of data and potential relationships [file 12889_2023_16259_MOESM4_ESM.pdf]

**Additional file 4**

**Co-occurrence of data and potential relationships**

| **No.** | **Co-occurrence code** | **Meaning units** | **Interpretation** |
|---|---|---|---|
| 1 | Lack of family support AND income insecurity AND cost of transportation | *"I live alone at Obuasi and do not have anyone to help me so I had to leave for my hometown and only went back to the hospital whenever my medications got finished for another set of medication. However, it got to a time where all my money got finished, so I did not get money to take a vehicle t to the hospital."* – Male TB patient, 60 years old | Lack of family support leads to income insecurity and non-adherence to TB treatment due to the cost of transportation |
| 2 | Long distance to TB treatment center AND cost of transportation AND non-adherence to treatment | *"I stay far from the tuberculosis treatment center in Obuasi and had it not been for my sister who assisted me with my transportation, I would not have been able to come for my medications even up to the third month after which I stopped coming for the medication"* – Male TB patient 52 years old | Long distances to a treatment center are a cause of increased transportation costs and non-adherence to TB treatment. |
| 3 | Long distance to treatment center AND difficulty accessing public transport AND non-adherence to TB treatment | *"When I am coming to the hospital, I walk from Odumasi to Aboagyekrom, and pass behind a school to Boete where Bryant Mission Hospital is. It is very far. Walking to the treatment center is what disturbs me, it makes my breathing rapid. Because I walk, I stopped taking the drugs after two months when the cough seized."* – Male TB patient, 37 years | Long distances to a treatment center are a cause of difficulty accessing public transport and non-adherence to TB treatment. |
| 4 | Cost of transportation AND food insecurity | *"Money for feeding and transportation to the hospital are the reasons why I couldn't complete my TB treatment, but money to pick a vehicle to the hospital was my main challenge."* – Male TB patient, 31 years old | Transportation costs and food insecurity lead to non-adherence to TB treatment. |
| 5 | Knowledge about TB treatment AND non-adherence | *"...I did not know I was supposed to continue to take the drugs till the sixth month despite having seen the doctor, so for almost one month I never took any drug."* – Male TB patient, 35 years old. | Insufficient knowledge about TB treatment is a cause of non-adherence to TB treatment. |

| | | | |
|---|---|---|---|
| 6 | Negative perception about the health care system AND self-decision to discontinue treatment | *"When I defaulted, I felt shy and feared to return to the hospital because I thought the health workers will be angry and complain that I did come for my drugs on time so I decided not to come at all."* – Male TB patient, 37 years old | A negative perception of the health care system is a cause of the psychological problem and self-decision to discontinue treatment. |
| 7 | Side effects of drugs AND self-decision to discontinue treatment AND food insecurity | *"Taking the drugs makes me feel very hungry so I decided to stop taking them because I would not get money for food."* – Male TB patient, 52 years old | Treatment side effect leads to self-decision to discontinue treatment as a result of food insecurity |
| 8 | Income insecurity AND lack of family support | *"Because I am unwell and so I am unable to work, my wife had to ask for a divorce since I could not get money to cater for my family, so I did not get anyone to support me from then."* – Male TB patient, 52 years old | Income insecurity due to TB disease leads to a lack of family support |
| 9 | Income insecurity AND cost of transportation AND non-adherence to treatment | *"It was my brother who was giving me money for transportation but of late, because of financial constraints, he is not able to give me money to come for my medications when they get finished, so it is now that he has gone to borrow money for me to be able to come for my medications*." – Male TB patient, 60 years old | Income insecurity leads to non-adherence to TB treatment due to transportation cost |
| 10 | Income insecurity AND cost of transportation AND food insecurity AND delayed treatment | *"From Kwabenakwa Junction to Mampamhwe where I stay is GH₵2.50. Taking vehicles back and forth will be GH₵5.00. Now from Kwabenakwa Junction to Boete is GH₵1.80 and from Boete to the hospital is GH₵1.50. If I take a car from Mampamhwe to the treatment center and back to the house is more than GH₵10. I hardly get such an amount of money and I surely cannot go to the hospital on empty stomach. This is why I cannot visit the treatment center for my drugs according to schedule."* – Male TB patient, 41 years | Income insecurity leads to problems with transportation costs and food insecurity and causes delayed treatment. |

| | | | |
|---|---|---|---|
| 11 | Income insecurity AND food insecurity AND non-adherence to treatment | *"They said when I take the drug, I should wait for one hour before I eat. So, at a point when I don't have money to buy food, I don't take the drug. I only take the drug only when I know that I have gotten money to buy food to eat"* – Male TB patient, 65 years old | Income insecurity leads to non-adherence to TB drugs due to food insecurity |
| 12 | Income insecurity AND food insecurity AND non-adherence to treatment | *"They usually gave me drugs within two weeks intervals so when I start and it is left with about four or five days for it to finish and I don't have money to feed, then I break, so when in two days I get money then I drink it, and when it is finished, I go for continuation from the hospital. "*– Male TB patient, 37 years old | Income insecurity leads to non-adherence to TB drugs due to food insecurity |
| 13 | Income insecurity AND food insecurity | *"It is the hunger I was going through that made me stop. What I will use to pick a car to the hospital for my drugs, I will rather use it to buy food."* – Male TB patient, 33 years old | Income insecurity leads to food insecurity and non-adherence to TB treatment |
| 14 | Income insecurity AND lack of family support AND food insecurity AND non-adherence to treatment | *"I lost my job, and I have no one to take care of me and I find it difficult to get food to eat. When I take the drugs, I become very hungry. I was able to take half of the drug in the first month that I started but I couldn't continue because I did not get food to eat and support."* – Male TB patient, 32 years old. | Income insecurity leads to a lack of family support and food insecurity which subsequently causes non-adherence to TB treatment. |
